# Reflections of Foster Youth Engaging in the Co-Design of Digital Mental Health Technology: Duoethnography Study

**DOI:** 10.2196/53231

**Published:** 2025-01-20

**Authors:** Ifunanya Ezimora, Tylia Lundberg, Dylan Miars, Jeruel Trujeque, Ashley Papias, Margareth V Del Cid, Johanna B Folk, Marina Tolou-Shams

**Affiliations:** 1 Department of Psychiatry and Behavioral Sciences University of California, San Francisco San Francisco, CA United States

**Keywords:** foster youth, digital health technology, co-design, app development, mental health, adolescent, young adult, mobile health, mHealth, foster care, duoethnography

## Abstract

**Background:**

Current research on digital applications to support the mental health and well-being of foster youth is limited to theoretical applications for transition-aged foster youth and support platforms developed without intentional input from foster youth themselves. Centering the lived expertise of foster youth in digital solutions is crucial to dismantling barriers to care, leading to an increase in service access and improving mental health outcomes. Co-design centers the intended end users during the design process, creating a direct relationship between potential users and developers. This methodology holds promise for creating tools centered on foster youth, yet little is known about the co-design experience for foster youth. Understanding foster youth’s experience with co-design is crucial to identifying best practices, knowledge of which is currently limited.

**Objective:**

The aim of this paper is to reflect on the experiences of 4 foster youth involved in the co-design of FostrSpace, a mobile app designed through a collaboration among foster youth in the San Francisco Bay Area; clinicians and academics from the Juvenile Justice Behavioral Health research team at the University of California, San Francisco; and Chorus Innovations, a rapid technology development platform specializing in participatory design practices. Key recommendations for co-designing with foster youth were generated with reference to these reflections.

**Methods:**

A duoethnography study was conducted over a 1-month period with the 4 transition-aged former foster youth co-designers of FostrSpace via written reflections and a single in-person roundtable discussion. Reflections were coded and analyzed via reflexive thematic analysis.

**Results:**

In total, 4 main themes were identified from coding of the duoethnography reflections: power and control, resource navigation, building community and safe spaces, and identity. Themes of power and control and resource navigation highlighted the challenges FostrSpace co-designers experienced trying to access basic needs, support from caregivers, and mental health resources as foster youth and former foster youth. Discussions pertaining to building community and safe spaces highlighted the positive effect of foster youth communities on co-designers, and discussions related to identity revealed the complexities associated with understanding and embracing foster youth identity.

**Conclusions:**

This duoethnography study highlights the importance of centering the lived expertise of co-designers throughout the app development process. As the digital health field increasingly shifts toward using co-design methods to develop digital mental health technologies for underserved youth populations, we offer recommendations for researchers seeking to ethically and effectively engage youth co-designers. Actively reflecting throughout the co-design process, finding creative ways to engage in power-sharing practices to build community, and ensuring mutual benefit among co-designers are some of the recommended core components to address when co-designing behavioral health technologies for youth.

## Introduction

### Background

Over 391,000 youth are in foster care in the United States, with behavioral health being their largest unmet need [1]. Foster youth are young people removed from their caregivers due to safety risks or unforeseen circumstances (eg, death of caregivers) and placed in out-of-home care that aims to be safe and stable. Out-of-home placement, such as group homes and placement with a foster parent, can precede reunification with their original caregiver, permanent placement with a legal guardian or adoptive family, or other planned permanency arrangements [2]. Contact with the foster care system at key periods of development and abrupt placement with new caregivers can contribute to foster youth experiencing adverse behavioral health outcomes, increased risk of not having an accountable caregiver, and insufficient access to services that support their well-being [3]. Changes in placement can result in service disruption, further exacerbating unmet mental health needs. However, digital health technology may provide an intermediary behavioral health support solution while youth transition between placements or act as a longer-term support that can go with them anywhere from placement to placement. There is a need to target widespread inequities in behavioral health service access and involve foster youth voices in the development of new resources. Currently, there is a gap in the literature on the development, deployment, and evaluation of mobile apps designed for foster youth. Foster youth have been excluded from conversations regarding tools designed to support their well-being despite their lived expertise being crucial in targeting barriers that drive adverse mental health outcomes and underuse of services [3]. Co-design holds promise for creating youth-centered tools that are responsive to the needs of specific communities such as foster youth. Despite the potential benefits, little is known about how best to facilitate co-design with youth from underserved groups, such as foster youth, or their experiences working on co-design teams.

### Co-Designing Mobile Apps

Interventions to address mental health and resource needs have increasingly shifted toward digital health technologies. Digital health methods hold great potential for addressing gaps in services for underserved communities, and using co-design methods may better meet the needs of these communities [4]. Co-design processes create a direct relationship between potential users and designers, where intended users from target communities actively participate in designing technologies for their community [4,5]. Co-design centers users in the design process and lies at one end of the user-centered design spectrum. Co-design processes with young people are meant to center youth involvement throughout the design process, but many technologies claiming to use co-design processes with youth use the term *co-design* to reference a continuum of user involvement ranging from minimal youth involvement at the level of consultation to more integrated involvement through mutual partnership (true co-design) [4].

Recent co-designed applications include mood-monitoring applications for youth experiencing depression; integrated digital crisis planning tools for youth; personal informatics apps; and open access, anonymous platforms with evidence-based, self-guided interventions for youth mental health [6-9]. Co-designed digital health technologies for both youth and adults have used human-centered co-design with studio methodology (ie, an iterative process of young people sketching out an idealized app and its features), adolescent-led and initiated co-design processes at a higher level in the “ladder of participation,” and participatory technology development incorporating community-partnered research principles with technology platforms (eg, Chorus) [6,8,10,11].

Researchers have begun designing digital health technologies with greater intentionality in their work with young people by including them as early in the research design process as possible [5,12] and have called on others to do the same. However, there is still a dearth of mobile apps designed by young people for young people. Current data estimate that >10,000 existing mental health apps are available for download [13]. However, a 2020 systematic review identified only 30 mental health technologies that were co-designed digital mental health technologies for children and young people [4]. Another systematic review in 2020 surfaced only 10 publications specifically on mobile apps and web applications for young people, with most being focused on the dissemination of mental health information to specific populations [14]. With the increased use of co-design for the development of web-based mental health resources and the lack of literature on co-design with foster youth, it is imperative to focus efforts on pinpointing strategies that help center the voices of the intended beneficiaries in the creation of these technologies.

Apps and web-based platforms designed to be used by the foster youth community (and caregivers) exist. For example, FosterClub and iFoster are 2 web-based communities targeted toward empowering current and former foster youth through resources and opportunities [15,16]. Currently, iFoster is led by a former foster youth with team members with lived expertise working with children in care and transition-aged youth. FosterClub was founded by a caregiver of 2 foster youth to “help fill the gap she perceived her boys—and other children and youth in foster care in America—were experiencing: a lack of access to a peer support group and information to help them navigate the foster care system” [16]. However, these websites were designed and created by resource providers and professionals (some of whom were former foster youth that had long aged out of the system) and did not directly partner with current or transition-aged foster youth to address the emotional wellness needs of the foster youth community at creation. A recent study with current and former foster youth analyzed a conceptual framework for developing a mobile app to assist youth in transitioning from foster care to adulthood; however, this study did not result in the formal creation of the app [17]. Thus, there remains a need to center the lived expertise of young adults presently in or recently aged out of the foster care system in designing and building emotional wellness tools. Particularly in this era defined by technology and increased accessibility, there is no room to exclude minoritized young people, such as foster youth, from participating in the creation of digital health technology meant to support them.

### FostrSpace

FostrSpace is a mobile app designed and created through a collaboration among San Francisco Bay Area foster youth; clinicians and academics from the Juvenile Justice Behavioral Health (JJBH) research team at the University of California, San Francisco (UCSF); and Chorus Innovations, a rapid technology development platform specializing in participatory design practices. FostrSpace was created to take the stigma out of accessing mental health care and resources for young people who have been in foster care by offering access to digital psychoeducational materials, a personal resource navigator, and licensed clinicians who provide tele–mental health services. To our knowledge, FostrSpace is the only mobile app focused on improving emotional wellness resources for the foster youth community that was co-designed from inception to production to iteration with foster youth. FostrSpace is currently live and can be accessed at its website [18].

### Aim of This Study

The aim of this paper is to reflect on the co-design experience of the 4 foster youth involved in the development of FostrSpace. The focus of this manuscript is not on the methodology of co-design as a practice but, rather, on the foster youth experience participating in the co-design process, including how lived experience in the foster care system informed what was cocreated and how the co-design process impacted the foster youth co-designers. These reflections are later leveraged to provide recommended practices for academics aiming to facilitate effective co-design spaces with foster youth. The preceding section briefly outlined the FostrSpace app and its features. The aim of this manuscript is to offer insights into the co-design process from the perspective of foster youth. Rather than providing an exhaustive description or outcomes of the resulting app, our emphasis remains on reflecting on the collaborative design experience. The FostrSpace co-design process reflects the critical duty of academics to collaborate with minoritized communities when developing technological resources. In general, academics without lived expertise in the foster care system can be at higher risk of recreating experiences that result in mental health and resource disparities or creating web-based spaces that exacerbate trauma experienced by foster youth. The duoethnography practice used in this study built on research by Porche et al [5] that aimed to promote more authentic partnerships between academics and minoritized communities. The ultimate goal is to dismantle barriers to resource access while nurturing mutually beneficial relationships among all participants. Through the duoethnography methodology, Porche et al [5] focused on the reflections of 4 psychologists and psychiatrists in academic medicine, 2 of whom are also authors of this paper. Porche et al [5] offer broad co-design reflections across various projects (including FostrSpace), highlighting barriers and challenges in sharing co-design responsibility with co-design partners. The paper by Porche et al [5] focuses solely on reflections from academic co-designers and does not include perspectives from community co-designers. This paper expands on the work by Porche et al [5] by examining the co-design process from the youth perspective and specifically with foster youth. The use of duoethnography in examining the FostrSpace co-design process helped reveal candid reflections on foster youth’s lived expertise and involvement in co-design. These reflections underscore how positive dynamics within the co-design team led to an open and collaborative partnership.

## Methods

### Co-Design

The design of FostrSpace used a community-partnered participatory co-design process to ensure power sharing between co-designers. Techquity, the process of achieving health equity through strategic development and deployment of technology, guided the co-design [19]. Digital health technologies can help connect young people with care, addressing service use deficits and cost barriers. Techquity applies a critical lens to the inclusion and exclusion of individuals during the technology creation process, recognizing that excluding certain voices leads to gaps in technological advancements.

The co-design process used youth participatory action research (YPAR) principles with an antiracist framework. YPAR challenges assumptions about who is allowed to conduct research, placing young people in a central role to investigate social conditions (eg, the foster care system) that impact their lives [20]. YPAR principles underscore the significance of establishing a secure and inclusive environment for involving young people in research processes. Our commitment to safety and inclusion was manifested through practices such as ensuring that participants could make informed decisions about their engagement, highlighting voluntary participation, upholding the confidentiality of shared information, fostering active involvement in decision-making, and implementing continuous feedback and reflection mechanisms. Similarly, we were mindful of racialized research positionality and cultural and developmental contexts, deferring to the FostrSpace Advisory Board (FAB)—a name chosen by the members—co-designers for language and framing feedback whenever possible on the app. YPAR is a valuable approach to support young people wanting to make a positive impact, proving especially impactful for those facing marginalization rooted in racism, sexism, homophobia, transphobia, classism, ableism, or other oppressive factors. With this in mind, we chose YPAR as a framework to underscore the expertise of our foster youth co-designers and carefully manage power dynamics to promote power sharing. While all research projects within the JJBH research team incorporate antiracist principles, it requires a deliberate and sustained effort and, thus, was an intentional focus in the FostrSpace co-design. Particular efforts were made to value multiple ways of knowing and honor foster youth similar and dissimilar lived expertise, as well as counter paternalism to ensure that participating foster youth were empowered to direct FostrSpace’s creation from design to mechanics.

FostrSpace co-design included transition-aged foster youth, clinicians, academics, and technology developers in the same co-design space rather than holding separate parallel co-design processes, as is common in app development. By having all co-designers in the room, everyone could learn from one another and collaborate to integrate distinct experiences into the FostrSpace design process. Youth co-designers came with lived expertise and histories of foster youth advocacy work, whereas academic co-designers came with experience of co-designing with young people in other research projects [5]. Clinicians, some of whom held dual roles as academics, possessed clinical expertise in supporting youth well-being and facilitating group processes. Technology developers came with expertise creating digital health technologies through participatory design.

Co-design of FostrSpace started in the planning stages, with young people aged 16 to 26 years who were previously or currently involved in the foster care system being invited to participate in idea-sharing work groups. The work groups were advertised through organizations and resource providers that have ongoing collaborations with the JJBH research team (eg, the San Francisco Unified School District), who shared recruitment flyers within their networks. In total, 2 work groups took place in September 2021, qualitatively exploring how a mobile app could help support foster youth in identifying and navigating mental health and wellness resources, supports, and interventions. The first work group had 3 participants, and the second work group had 2 participants; each work group lasted 90 minutes and was held with a different group of foster youth. A call was put out to the 5 participants over the 2 work groups to join the co-design team in a more formal staff capacity. Once the team was finalized (ie, the FAB members were hired), formal co-design meetings for FostrSpace took place on a weekly basis.

### Duoethnography

#### Overview

Duoethnography, as defined by Sawyer and Norris [21], was used to guide the analysis. This research method aims to look at “the cultural contexts of autobiographical experiences in order to gain insight into their current perspectives and experiences of issues related to personal and professional identities” [22], allowing researchers to use their “life curriculum” to collaboratively examine and apply meaning to the experience of interest. In this context, the duoethnography process focuses on the experience of 4 self-identified foster youth (the FAB members) who codeveloped the mental wellness app FostrSpace with a team of academics, clinicians, and technology developers. Duoethnography was regarded as the most appropriate approach for this study as it allowed for centering the voices of the FAB. Furthermore, the duoethnography reflection process promoted further collaboration between the FAB and the academic co-design team. The FAB’s sharing of their lived experiences that motivated involvement in FostrSpace highlighted the importance of having young people with lived expertise contribute to interventions aimed at their community, as well as the practices that were considered to have made the co-design of FostrSpace a productive, reflective space.

#### Participants

Coauthors of this paper include 4 FAB co-designers (TL, DM, JT, and AP), with whom the duoethnography was performed, and 4 academic co-designers (IE, JBF, MVDC, and MT-S), who did not provide reflections for the duoethnography. As part of the duoethnography process, FAB co-designers provided both written reflections and verbal responses via a roundtable discussion regarding their experiences co-designing and cobuilding FostrSpace. All FAB members also contributed to the writing of the duoethnography in a multitude of ways (eg, co–first author and author) through providing data to shape the thematic analysis (TA), being involved in TA (eg, coding and writing), or providing feedback on the themes derived (eg, whether quotes were used appropriately or themes were derived appropriately). The collaborative academic co-designers and authors (n=4) were involved in providing structure to the co-design of the FostrSpace app, the reflection prompts, and the roundtable discussion. All academic co-designers were involved in the writing of this manuscript, in TA (eg, coding and writing), or in providing feedback on the themes derived. While the sample size of 4 FAB members contributing to the data collected for the TA is small, it reflects the number of young people with lived expertise in foster care who contributed to the development of FostrSpace and their reflections on the co-design process. Duoethnography does not have a set number of collaborators needed to engage in the methodology, rather focusing on the ability to maintain “intimacy, trust, and commitment” [22] to the tenets of duoethnography (eg, self-reflexivity) among 2 or more researchers. Similarly, reflexive TA dictates that the participant sample size for analysis depends on the size and scope of the project [23]. The FAB members’ reflections provided a basis for engaging with foster youth in collaborative development of digital solutions and a framework for centering lived expertise in the development of and research on mental health technology.

The FAB included a White nonbinary foster youth, a White-passing mixed Asian-Hispanic foster youth, a Mexican-Latino foster youth, and a Chicana foster youth. The FAB members are a group of current and recently graduated multilingual, first-generation college students who, together, have experiences in both the traditional foster care system and kinship or relative care, making them intimately aware of the disparities present in the child welfare system. At the time of the duoethnography, 75% (3/4) of the FAB members identified as former foster youth, and 25% (1/4) identified as transition-aged foster youth; they maintained a consensus that they were not current foster youth due to being aged over 18 years but were still impacted by the identity. Through their upbringings, FAB members experienced poverty, low-income, working-class, and middle-class statuses, living in group homes, kinship caregiver households, community housing centers, campus housing, and individual housing. As a group, they hold both cisgender and queer identities, various levels of connection and separation from their cultural identities, different levels of disability status, and part-time positions in both FostrSpace and other jobs in the community while maintaining statuses as students or full-time employees.

The academic co-designers included a first-generation Nigerian Black pangender and pansexual research coordinator; a first-generation Guatemalan American, neurodivergent, cisgender, and straight light-skinned Latina clinical psychologist; a White Jewish genderqueer and gay clinical psychologist; and a White-passing Iranian immigrant cisgender and heterosexual clinical psychologist. All are conducting community-engaged research to increase behavioral health service access for youth and families in contact with the juvenile legal and child welfare systems and have had personal ties to these communities.

Through connected but distinct lived expertise and various roles that we occupy in community and academic spheres, all the coauthors of the paper have an awareness of the types of hardship that foster youth can experience from childhood to adulthood and how those hardships interact with intersectional identities. These hardships included social and emotional factors such as parentification from a young age as well as structural and systemic barriers to resource navigation in the behavioral health, medical, and educational spheres. Our own identities and lived expertise drive us to address these barriers to improve conditions for foster youth and provide a lens for interpreting our results.

#### Procedure

Following duoethnography guidelines, FAB members reflected on their experiences in the foster care system and how this related to their experience in the FostrSpace app development co-design process (eg, what they personally got out of the process and how they conceptualized different features on the app) via written reflections and verbal discussions. Members spent 1 month (April 2022-May 2022) writing their own reflections and reading and responding to their fellow FAB members’ reflections. Following the writing period, an in-person roundtable discussion about the co-design experience was held to allow members to verbally share their reflections as well (eg, because not all co-designers felt as comfortable with written reflections). The 4 FAB members engaged in an hour-long discussion with JBF as a facilitator and IE as notetaker. The facilitator and notetaker were academic co-designers that contributed to the FostrSpace app development and duoethnography paper writing; neither held a formal supervisory role over the FAB members (ie, the supervisor on record was not involved in the duoethnography process). We acknowledge that the power dynamics of having academic co-designers facilitate the roundtable discussion among the FAB members about their co-design experience could have impacted what was and was not shared. Keeping this in mind, academic co-designers provided structure to the discussion through the prompting of discussion questions while allowing space for the co-designers to talk with one another freely on the topics with limited interjection. In addition, FAB members and academic co-designers built mutual, trusting partnerships with one another during the co-design process, and the duoethnography took place after the bulk of the co-design process was concluded and the app was functional. As a result of the mitigation of the potential consequences of negative power dynamics through these methods, FAB members felt comfortable sharing sensitive data in the roundtable, as indicated by the vulnerability and honesty exhibited in the quotes used in the results as well as within co-design meeting discussions and one-on-one check-ins with staff. Reflection prompts were collaboratively developed by the coauthors before starting the duoethnography process, keeping in mind the research aim of understanding the experience of foster youth co-designing an app in collaboration with academics, clinicians, and technology developers. The same prompts were used for the written and verbal reflections. The prompts are available in Textbox 1.

**Reflection prompts used in the FostrSpace duoethnography with FostrSpace Advisory Board co-designers over the 1-month reflection period.**

- Why did you get involved in this process? What encouraged you to keep going? After attending the first meeting, what kept you going to the second, third, and so on?
- How do you feel about the way the co-design was handled? In what ways would you have liked the process to go differently? What were your experiences of power dynamics in the co-design process and in what you bring to this work?
- Why is it important to center lived experience in this work? What parts of yourself and your experiences in foster care did you bring into this work? What inspired you throughout your time in foster care to have interest in what we are doing? What support did you lack in foster care? Remember: not every story is the same—not everyone would do the same things growing up and everyone’s feelings are valid.
- How have you changed throughout the course of the co-design process? How have your goals or aspirations or mindset changed since starting this project?
- Has this project amplified your voice or feelings toward reform within the foster care system? In what ways?
- Why do you think this is a good resource for other foster youth to have?

#### Data Analysis

Reflexive TA [23-27] was used with 9 pieces (n=8, 89% written reflections and peer responses to reflections and n=1, 11% roundtable transcription) from the 4 FAB members. Reflexive TA was specifically chosen (as opposed to a coding reliability TA or a codebook approach to TA) to be congruent with the co-design value of honoring participant lived expertise. Reflexive TA allows for the analysis of participant subjective data through the lens of researcher reflexive interpretations [26]. An inductive, “bottom up” approach under an essentialist and realist paradigm was used by the first author coders (IE and TL) to ensure that identified patterns within the data were pulled from the data itself [23-27]. Initial codes aimed to provide a rich description of the full dataset to capture the breadth of factors that should be at the forefront of academics’ minds when co-designing with individuals with lived expertise in the foster care system. Themes were developed using a semantic approach in which surface meanings of the data were focused on in the initial coding process. This distinction was important for the coders as a way to acknowledge the prevalent history in the foster youth community of feeling disempowered by individuals and groups in power. By using a semantic approach to theme development, coders hoped that the process would be more empowering and respectful to the FAB members’ experiences. A realist and essentialist paradigm rather than a constructionist approach was used to focus on individual psychologies as well as differences and similarities in lived expertise among the FAB members.

In line with the 6 guidelines by Braun and Clarke [23-27] for reflexive TA, the coders first familiarized themselves with the dataset before initiating coding. As an FAB member and participant in the verbal discussion and written prompts and reflections, TL was intimately familiar with the dataset. Similarly, as lead research coordinator on FostrSpace, IE read the FAB reflections as they were being written and acted as the notetaker during the roundtable discussion. All qualitative analysis was conducted in ATLAS.ti (ATLAS.ti Scientific Software Development GmbH) [28]. The 2 coders independently generated initial codes related to the co-design process and the foster youth experience. Preliminary codes were discussed collaboratively among the coders and coauthors (IE, TL, JBF, and MT-S) to develop a final list of codes. A total of 18 codes (initial broad themes) were developed and used to conduct final coding through which themes were developed (Textbox 2). Analysis involved more than one coder and, therefore, necessitated collaborative discussion.

**The 18 codes developed from thematic analysis and applied to the 4 FostrSpace Advisory Board members’ reflections on the co-design process.**

**Codes**

- App as a place for transparency
- App as a safe space
- Barriers to accessing resources that are not foster youth specific
- Barriers to accessing resources specifically for foster youth
- Co-design as a safe collaborative space
- Future goals for the app
- Identity (negative)—external gatekeeping
- Identity (negative)—internal gatekeeping
- Identity (positive)—finding, accepting, and embracing
- Initial app involvement
- Intersectionality
- Lack of clarity—foster care system
- Motivation for the app—paying it forward
- Negative experiences in kinship care
- Positive community support
- Positive impact of co-design participation on the self
- Resource stigma
- Stigma toward foster youth

The results include final analyses of the selected extracts and relevant themes, which were reviewed and approved by the coauthors of this paper, including the entire FAB team, who had the option of retracting extracts of their data that they did not feel comfortable sharing with the public. All FAB members endorsed the information presented in the manuscript and opted not to retract data.

### Ethical Considerations

The institutional review board of the UCSF determined that this research is exempt from institutional review board oversight according to the federal regulations summarized in Title 45 of the Code of Federal Regulations Part 46.102(l). Participants from the work groups were compensated with a US $75 gift card for their participation. A total of 80% (4/5) of the individuals from the work groups applied for and joined the final co-design team, making up the FAB. FAB members were then hired as UCSF employees to co-design the FostrSpace app and were compensated at an hourly rate based on their individual compensation package, which was determined by educational level and experience as set by UCSF Human Resources for all the time spent completing tasks related to UCSF employment (eg, co-designing the FostrSpace app and engaging in the duoethnography reflections). The FAB as employees of UCSF must abide by federal and state employment laws as well as have access to human resources to support any workplace issues.

## Results

### Overview

Coders derived 4 main themes from organizing codes around core commonalities through reflexive TA. The final themes addressed the experience of taking part in the co-design process, including the FAB members’ broad experiences in the foster care system, which directly influenced their approach to and navigation through the co-design process and app development. The number of quotes presented in the results from each FAB member ranged from 5 to 15 excerpts. Themes included (1) power and control (loss of power, reclamation of power, and power dynamics), (2) resource navigation, (3) building community and safe spaces, and (4) identity. All themes were rooted in explicit content from the coded data.

### Power and Control

#### Loss of Power

FAB members described their experiences with a loss of power due to the nature of their foster youth status through childhood, adolescence, and even extending into young adulthood. Systems (ie, the child welfare system) allegedly designed to empower and protect them withheld crucial information necessary for FAB members to feel like they had agency over their own lives. To illustrate, the lack of transparency regarding monthly payments made to foster parents (both the monetary amount and transaction timeline) led to frustration and disempowerment stemming from FAB members feeling that they were left in the dark about their own care:

So, for a while, I was just like, “I’m homeless.” Or I’m not homeless, “I’m poor.” I was just like, “I’m poor. I can’t ask for anything.” And then 10-plus years later to find out that that person was still getting paid to take care of me? And I’m like—because they’re family, you know what I mean, it’s [sic] was like they didn’t really owe you much?

FAB member 3

Existing foster care systems and policies (eg, foster youth not having a say in placement with family members) constantly take away power and control from foster youth, making decisions for them without their input. Decisions made by guardians without FAB members’ input negatively impacted the ability of FAB members to access vital information necessary for their care:

I asked my grandparents, “Who is my social worker?” They were like, “Oh, we shredded all those documents because it didn’t apply because they weren’t coming anymore. And we were good.” I’m like, “What?” So, I tried calling the county, they never got back to me. They just put me in this whole rigamarole.

FAB member 2

In addition, though part of a larger network of youth in the child welfare system, FAB members noted the isolation prevalent in the foster care system:

Growing up foster kids were never given a platform to speak and ask for help. I personally was never given resources growing up that could help connect me to other foster youth or provide mental health resources when I needed them.

FAB member 1

#### Reclamation of Power

By participating in the co-design of FostrSpace, FAB members were able to reclaim some of the power they had lost and their decision-making ability. Members felt empowered to reclaim control over their own trajectories as well as lay the groundwork for foster youth coming after them to assert agency. Through FostrSpace, FAB members felt that they could equip current and future generations of foster youth with information and resources, which could allow them to take control of their own lives and mental health:

I agree that the app is a great place for current and past foster youth to find a supportive community to share with as it is not always easy to reach out to the people around you for help. Especially when you’re searching for a safe space to share struggles with mental health as so much stigma still surrounds mental illness in the United States as well as being a former or current foster youth.

FAB member 4

In addition to promoting resource access, co-designing FostrSpace strengthened FAB members’ feelings of power and control by educating youth about the foster care system. Growing up in the isolation of the foster care system led to increased appreciation for the transparency available in FostrSpace:

[My caregiver] villainized everybody and it was like we cut contact off with everyone. It was really like isolating. So, I like that we have this resource where we can go search it up and be like, “Okay, well, what does it mean to be a foster kid? Where can I find a resource to go get some money for food?”

FAB member 1

FAB members emphasized the importance of giving users ample information to equip them with knowledge, encouraging them to be informed consumers. FAB members appreciated how FostrSpace leans toward having too much rather than too little information:

That’s the thing—like the app. I remember I always like where we specify everything down to a T. Sometimes it gets a little wordy and that’s annoying for some users of the app, but it explains everything to you clearly.

FAB member 1

With the opportunity to use their lived expertise in the development of the web-based platform, FAB members felt that they could reclaim the narrative over their life. FostrSpace gave them the opportunity to regain control after years of experiencing intentional withholding of control by the system:

It really points to just this greater idea of mitigating suffering. This greater idea of moving beyond and I think that’s really important. I’ve always been focused on this as a greater abstract idea, but this is one of the ways I’ve actually nailed down providing support and making the world better, in a limited way that I can. And so, I’ve really appreciated having the space to allow me to actually do this.

FAB member 2

#### Power Dynamics

The interplay of power dynamics was key for FAB members regarding life experiences as detrimental versus supportive. Harmful power dynamics manifested in both navigating governmental assistance and the family unit. While navigating already mystifying systems, FAB members had to advocate for themselves to individuals in positions of power when submitting applications for food assistance, housing, and more. FAB members also expressed feeling as if their own realities and experiences were minimized or denied when trying to access resources:

...I had to call back for my EBT [electronic benefits transfer] because they denied it last month. I remember calling them and I was like, “Hey, you guys denied it. Even the app says I didn’t have any money entered.” And she’s checking her records and she’s like, “No. We put it in.” And I was like, “Lady, like no you didn’t.”...I remember just being like, Come on. I’m already telling you I had to use money out of my own pocket that I’ve been trying to save for rent. I know what I’m using my money for.

FAB member 1

Government and school staff alike also denied FAB members’ foster youth identities and ability to access foster youth–specific resources because they did not have proper documentation labeling them as foster youth:

In school, I remember I was constantly denied. I asked if they could get help finding foster youth resources and scholarships, and I literally had staff tell me, “You’re not a foster kid. I don’t have documentation.”

FAB member 1

Consistently facing roadblocks and antagonism from the systems designed to help them led to feelings of disempowerment among the FAB members. Having to go up the chain of command to receive a response or going through several layers of forms, applications, and paperwork only for these to be rejected accumulated experiences of trauma and negatively impacted the motivation to access these resources for some FAB members:

I am and have been eligible for EBT [electronic benefits transfer], but after doing it for a year I don’t do it...how many of these are actually foster youth-friendly? Because we can tell people to sign up for SNAP, but when we look at these programs the blatant villainization or willful ignorance to what we’ve gone through and the struggles we are going through makes it so it’s harder to reach out for these resources...they don’t take me seriously when I tell them about the trauma that comes from it.

FAB member 2

FAB members experienced kinship care as mirroring the unsupportive power dynamics in governmental assistance, with kinship care “family” behaving differently from how a “traditional” family or chosen family or community may be thought to behave. FAB members described feeling alienated due to the dynamics of being in a kinship care household:

...even though foster youth may have access to family in foster care through family members being their foster parent or having their sibling with them, it’s still easy to feel alone. I personally felt that as the oldest sibling of three I couldn’t be vulnerable when I was struggling as everyone else was also having a difficult time. In my experience being in foster care made it difficult for me to ask for support, I was uncomfortable being in a new parental dynamic with people I didn’t know too well so it was hard to ask for things.

FAB member 4

FAB members experienced kinship caregivers withholding resources and information about the foster care system that FAB members needed even though they were meant to be trusted adults who could provide care:

She just wanted to do stuff where again she was the only one in control. And with me going and finding these resources? She didn’t help me go do that. She was leaving me on my own and like, “Go do that.” Again, it’s just like she’s leaving me out there and alone. It was really sad.

FAB member 1

Similarly, FAB members felt the weight of being the different party in a new caregiver-youth dynamic with mixed signals:

But looking back on [growing up], it was a heavy load kind of me just to like—well, my family is proud of me? No, they’re not proud of me because I’m smarter than I guess they thought I’d be. So, for a while, it was just like wow. I was just so conflicted where it was like, “Am I doing the right thing? Should I keep pursuing my education?”...I’m like...“Why are you talking down on me and making me feel bad?...Isn't this what y'all wanted for me?”

FAB member 3

The ability of the co-design process to empower FAB members was in great part due to the intentional way in which power dynamics were attended to in the process:

I remember telling everybody like, “Dude, it’s equal here...they don’t talk on top of each other. They don’t cut you off. They let you finish your idea even if it’s dumb because it might be.” [laughs]...here everyone is on an equal playing field, even your bosses. They’re all chill with you...they’re always, they’re engaged. They want to listen, they want to do something, and they want to help the community. I think that’s the most important part is the want to do something and then actually doing it.

FAB member 1

Notably, as individuals who had experienced systems designed to uplift them repeatedly ignoring their voices, FAB members went into FostrSpace without hopeful expectations:

I didn’t necessarily anticipate having a big role in the development of this app, but I also didn’t have many expectations about its potential either. I became ecstatic when I received my official offer letter to join UCSF and begin the process of developing an app as an ambassador.

FAB member 3

Over time in the co-design space, FAB members became comfortable, learning that the participating academics were working for the betterment of the community and uplifting individual voices within the group:

...the co-design team was wonderful to work with as it felt all the foster youth giving feedback were heard and treated as equals by superiors. Overall, the friendly space that was created by the entire team during meetings and the positive influence the finished app would have in the foster youth community keep me returning to the meetings.

FAB member 4

### Resource Navigation

FAB members shared situational factors that contributed to inaccessibility of resources, including lack of knowledge of available resources and how to access them, responsive resources, adult figures to guide them toward resources, and documentation. FAB members expressed frustration over being stuck in a system with ambiguous directions:

Yes. Oh, my god. [collective agreement] All the [redacted name] county forms are like, “Worker ID this, ID this.” Then you're like, okay, who do I call? Leaving voicemails for the workers because they never want to answer their phones. Then they never call you back. It's just stuff like that where you're just sitting there like, “Okay, well, what do I do?”

FAB member 1

Furthermore, FAB members mentioned that, when they did access resources, these were often not responsive to their lived expertise and status as foster youth, instead following a one-size-fits-all structure. FAB members recounted situations in which resources did not meet their needs due to limitations in access (eg, time frame):

I remember telling my whole life story at five years old to this lady and just telling her, “Oh, this is what happened,” again the gory situation and everything, and she helped me cope with it. It was really good. I would walk out feeling refreshed. I felt like everything was just off my shoulders. Then after maybe like a few sessions it just stopped. And then I never got any resources.

FAB member 1

A lack of reliable adult figures during key periods of development was a common experience for FAB members, who suddenly lost support people who could direct them to needed resources. FAB members expressed that FostrSpace resonated with them as it attempts to connect foster youth with resources for mental health and basic needs:

 I had received an email about outreach being done through UCSF to develop an app to better support the needs of foster youth, particular in the field of mental health and connecting youth to basic needs. I was previously in foster care in my early teens and lost both my parents in my teens, these experiences made me feel like I had no adult guidance to show me what resources I had available to me as well as support me when I was dealing with mental health issues.

FAB member 4

When resources required FAB members to provide documentation (eg, case IDs, permanent address, or social worker’s name) that they did not have access to, they were hindered in submitting applications:

...they were doing scholarships or support funds for foster youth, and even like transition age. I remember I applied to it and they were like, “What’s your social worker’s name? What’s your case number?” All this stuff. And I didn’t have that information.

FAB member 2

FAB members also had difficulty identifying resources that they were entitled to as foster youth, such as Court Appointed Special Advocate workers:

Like, I’m going to be honest. I don’t think I ever had a CASA person. So, just like you guys talking about CASA, it’s like there’s so many different aspects of just being a foster youth where we all have different experiences.

FAB member 3

The resource navigation challenges experienced by FAB members were described in contrast to the FostrSpace approach, which could benefit foster youth experiencing the same deterrents to resource access. The web-based platform provides foster youth with a directory of resources and live help on emotional wellness or resource navigation without the need for documentation. Due to FostrSpace’s more straightforward approach to resource navigation, FAB members expressed that FostrSpace helps reinforce self-advocacy and facilitates positive resource navigation experiences:

Moreover, having good experiences accessing resources should further encourage foster youth to engage with resources as I have experienced and seen people turn away from help when their experience is negative.

FAB member 4

### Building Community and Safe Spaces

FAB members discussed the importance of centering building community and safe spaces for foster youth. Membership in an established foster youth community positively impacted FAB members:

But I remembered when I got to UC Berkeley, my brother immediately was like, “Oh, my god, there’s this program called Berkeley Hope Scholars”... I signed up—and [Director name redacted] like a week before was just like, “Hey, this is what we have. These are the resources. We’re going to help you with everything.” And I immediately felt a sense of just comfortability. I felt safe there and I finally found a community where I could find other people who are like me or who experienced some of the same stuff as me.

FAB member 1

Similarly, the FAB community was beneficial for creating a space to collaboratively give back to the foster youth community through FostrSpace:

After attending the first meeting and meeting my co-workers I instantly felt as if I had found a great community of people fighting for change for a community that is often hidden in the shadows. I was excited to finally have my voice heard and to speak for my friends who experienced the system in a multitude of ways.

FAB member 1

There was shared comradery among FAB members as FostrSpace allowed them to unite under a common goal: to curate a safe space for heterogeneous foster youth tied by a common thread of lived expertise:

We’re dedicated to just creating a space so that people don’t have to further suffer the way we might have already, or in ways that we may not have experienced, but we don’t want anyone to ever risk going through.

FAB member 2

The importance of considering intersectionality was highlighted when building community and safe spaces for foster youth as foster youth experiences are shaped by how multitudes of identities, global world views, and inclusion interact. Through this duoethnography process, FAB members came to the realization that future iterations of FostrSpace’s resource directory need to take into account other identities that foster youth may hold (eg, race and gender) to better target nuances of resource inaccessibility:

...I’m always talking about my multitude of identities and intersecting identities. But that’s something where I don’t want resources just to default onto foster youth because sometimes they’re for White foster youth, Asian foster youth, or Black foster youth. It’s like specific. So, I’d really like to find more resources for trans youth, and for other races, as well, like Black, White, Asian, Latino—because there’s a huge Latino population in California. But just stuff like that where it’s resources for women’s reproductive health because again, I’m a woman and that’s something I have to go through...Like just where it covers those identities and takes care of kids that not only align under foster youth, but are foster youth AND, you know foster youth-included.

FAB member 1

FAB members also felt fortunate to have found a foster youth community as well as a common desire to develop a community within FostrSpace, expressing that FostrSpace could help connect more foster youth with a supportive community:

But then when I actually saw what y'all were doing, I was really moved by it. Like providing mental health and wellness resources for like—a I just felt like it spoke to an earlier me that was in desperate need for those and needed support and a community aspect.

FAB member 2

### Identity

FAB members engaged in rich discussions regarding the decision to identify as foster youth, the journey of coming to that decision, and the resulting consequences. Discussions highlighted that carrying a foster youth identity led to grappling with both positive and negative experiences. Within the context of negative experiences regarding identity, FAB members disclosed the strain that shame and stigma have on a foster youth’s ability to embrace their identity. Shame was described as something that was both ascribed by others (eg, strangers and family members) and internalized. FAB members associated the identity with traumatic upbringing:

I was never told that I was even a foster kid. When I said—like when me and my brothers said that name, my aunt would roll her eyes and be like, “What’s that? You’re just making stuff up. You’re dramatic.” So, it was like things like that. It had a very negative connotation around it.

FAB member 1

While kinship care is recognized as a part of the foster care system, FAB members noted internal shame regarding placement in kinship care:

I think part of it is because I still feel a bit of shame about my identity where there’s a part of me that’s just like, “Yeah, but you were raised by your grandparents. And even though you were in the system, do you really count?”

FAB member 2

In addition, they experienced external shame when actively aligning with the foster youth identity:

...when I created my website, I was really open about it, I’ve written a lot about it. Then one day, one of my distant uncles calls me. I’ll never forget this. I was getting ready and he was just like, “I saw that you posted an article on your Facebook about saying that you were a foster youth. You’re not a foster youth.” He was like, “How do you think your grandparents feel if you told them that you identify this way or that you say this?”

FAB member 2

Stigma was evident in the mischaracterization of the foster youth identity and pitying by others:

...when we were handing out flyers, one of my friends was like, “Watch out, they’re going to say sorry a lot.” I was like, “What do you mean?” And we were handing out flyers and the students were like, “Oh, I’m so sorry.” They’ll look at you with puppy-dog eyes and you’re just like, “I’m good. I’m chilling, bro. I just got some free lemonade.” [group laughter] It’s just stuff where you feel the name “foster youth”—just you feel like has this weight on it. And that’s what people add to it. It’s just like not like that.

FAB member 1

While negative associations with identity stemmed from general community (eg, schoolmates), family (eg, kinship caregivers), and internalized anti–foster youth dialogue from living with kinship caregivers, positive associations with their foster youth identity were built through engagement in the FostrSpace co-design process and with a foster youth community. Being a part of the co-design process offered FAB members space to reflect on negative experiences related to identity as well as build a new, positive association with the foster youth identity (eg, through community building in meetings):

...this process has...given [me] an ability to understand and process the uniqueness of what I have gone through, but also the commonality of the struggles and how no matter who you are, we all share these ideas, these feelings. And so why not try together to overcome them?

FAB member 2

Moreover, experience in an established foster youth community encouraged FAB members to embrace and accept identity, as well as providing a venue to receive support regarding struggles of identity. Co-designers expressed that being members of the FAB and working on the development of FostrSpace together validated their identity and allowed them to recognize that they were not isolated in their identity or experiences:

So, talking with you guys has always been great because every meeting is like, “Damn, I didn’t know that,” or, “Yes, I’ve been there.” Not that I’m glad that someone else has went through that with me, but it’s just like knowing that now collectively we can put that insight and experience to fruition.

FAB member 3

FostrSpace also acted as a platform to support the dismantling of stigma regarding the foster youth identity as FAB members were given the space to call on their lived expertise in the development of FostrSpace:

I felt as if I had just gained a new purpose in life, which was to share my lived experience and encourage others in the foster care system to embrace their stories and share it with pride.

FAB member 3

## Discussion

### Principal Findings

Co-design processes hold significant potential to create meaningful interventions for underserved populations such as current and former foster youth. The co-design process for FostrSpace exemplifies the value of centering the lived expertise of foster youth in the creation of digital tools designed to support them. The FAB members actively brought their own lived experiences navigating resources in their personal lives into the co-design space and used those experiences in developing the resource library for FostrSpace. As active co-designers, FAB members identified potential barriers to accessing resources that foster youth may face as well as brainstorming and problem-solving ways to inform the FostrSpace approach to resource navigation. For example, one barrier to accessing resources that FAB members identified was applications requiring details that foster youth may not have access to (ie, their social worker’s ID, particularly because some foster youth are not alerted to the fact that they have a social worker). Due to this, the FAB reflected as part of the co-design that FostrSpace needed to commit to providing resources made with foster youth in mind. Ideally, foster youth–tailored resources would not require information that foster youth do not have access to. To address this, the FAB recommended providing brief highlights on the information required to access certain resources (ie, CalFresh). This would equip foster youth with knowledge of potential barriers before trying to access the application. In line with this, during co-design, the FAB advocated for ensuring that foster youth did not face similar barriers and restrictions to accessing FostrSpace to those they faced in accessing other resources meant for foster youth (eg, as such, FostrSpace does not require foster youth to upload proof of status as a foster youth or social worker ID numbers).

Furthermore, reflections from the FAB members indicated that the co-design process was a safe and constructive space where all members worked together for the betterment of the wider foster youth community. As FAB member 3 stated, “Together, we can change the system and it all starts with collective effort of informing the broader community,” showing the drive to collectively work toward the shared goal of creating FostrSpace. This duoethnographic process adds to the literature of co-designing with young people and specifically to the scholarship of co-designing formal mental health applications with foster youth.

### Recommended Practices

#### Overview

The 4 main themes shared across the 4 FAB members demonstrate their reflections on their experiences with the FostrSpace co-design process, including motivations for participation and potential reasons why FAB members could not access the very resources they aimed to make more accessible through FostrSpace. From this reflection process, we recommend 3 key practices when co-designing with young people. Although we did not explicitly seek direct feedback from the FAB on these practices as part of the duoethnography process, the richness of the duoethnography data affirms the value of these practices in creating an enriching co-design space. In addition, while a number of different practices were brought into the FostrSpace co-design space, the key practices that we chose to list here are inspired by what was highlighted in the FAB members’ duoethnography quotes in addition to their endorsements in co-design meetings and check-ins. These recommendations are built from practices that were implemented in the creation of FostrSpace and are believed to have made the co-design process successful and worthwhile for co-designers:

1. Engage in active reflection on the co-design process both during and after co-design.
2. Find creative ways to engage in power-sharing practices to build community across diverse experiences and positions of power and privilege.
3. Ensure mutual benefit among co-designers.

#### Engage in Active Reflection of the Co-Design Process Both During and After Co-Design

Engaging in active reflection throughout the co-design process allows for the intervention (in this case, FostrSpace, a digital health technology) to become more actively shaped by thoughtful reflections. In the co-design of FostrSpace, brief icebreakers were used at the start of every meeting to ease individuals into lighthearted discussion before talking about a specific feature of the app. In addition, brief check-in questions were used at the end of meetings as well as in occasional one-on-one check-ins between FAB members and UCSF academic staff to evaluate co-designer comfort and feedback on the app. Although these engagement and reflection methods were highly useful in building community among the co-designers and allowing for free discussion, many of the reflections uncovered through the duoethnography were highly distinct and had not surfaced through previous reflection tools used during the co-design process. Specifically, by having a space designed solely for FAB co-designers to talk with one another and respond to questions not directly related to building the app through the in-person duoethnography process, we were able to reaffirm the most important features of the current app and important aspects to consider for iterations moving forward (eg, fully detailing a community element and the different ways in which this was crucial for this community).

Notably, in FostrSpace, we aimed to provide a robust resource directory for foster youth with food insecurity, legal or housing concerns, employment goals, or transportation goals, among others. We felt confident that we had developed strong resource lists within the counties that we initially served (eg, by tailoring resource pages with resource eligibility criteria, relevant website links, summaries, and office hours); however, we found through the duoethnography process that we were missing an essential element. The resources, though helpful when successfully acquired, often contained application or eligibility processes that were not foster youth friendly. This discovery, made possible only due to FAB members discussing their own personal frustrations with accessing public aid (eg, being given the “run-around,” having their needs denied, or being shamed for their needs) throughout the duoethnography, highlighted the need to seek out resource providers knowledgeable of the foster youth experience. It also reinforced the need to continue pushing the personal navigator to the forefront of the app as someone “who takes the role of goal-collaborator and accountabili-buddy by providing connections to resources so folks don’t have to navigate life alone” and ensuring that, for resources that are not foster youth friendly, app users have someone to support them throughout the process. Furthermore, FAB members’ experiences navigating public resources highlighted the importance of self-advocacy when dealing with resource providers lacking knowledge about the foster youth experience; personal and peer navigators (FAB members) can connect with users to help build self-advocacy to support resource access. FAB members as peer navigators and continued contributors to the app have since made content on sharing knowledge with current and former foster youth about skills they learned transitioning into adulthood, essentially creating a “how to” guide to accessing some common resources (eg, electronic benefits transfer).

#### Find Creative Ways to Engage in Power-Sharing Practices to Build Community

As noted previously, co-design meetings began with icebreakers, some led by UCSF academics, some led by Chorus designers and developers, and some led by the FAB members. Co-designers were encouraged to think of icebreakers in between meetings, topics they were curious about in their coworkers. Many came to the meetings excited to share their icebreakers, as FAB member 1 shared in the duoethnography roundtable:

I think the intro questions are my favorite part. I think that really showed it, too. They were like, “Hey, what’s your favorite taco spot?” It was just like small questions like that where you were like, Oh, okay, this is a cool space. Every time before each time, I’m like, Okay, what cool icebreaker questions will I ask my coworkers?

These icebreakers aided the development of a relaxed space before diving into the FostrSpace app design and development. All members had a chance to contribute and share something before transitioning into potentially daunting topics regarding designing an app. Furthermore, engaging in conversations about non–work-related topics served to shift the focus away from work titles and hierarchies, allowing participants to connect on a more personal level beyond the confines of the work environment. In addition, as co-design meetings were often in the early evening after a potentially draining work or school day for co-designers, the icebreakers helped in providing a check-in for co-designer energy levels, which influenced how many breaks were taken in a meeting and what agenda items were prioritized. FostrSpace was an app built through a co-design process that involved internal meetings among UCSF academics and clinicians; collaborative meetings with UCSF and Chorus designers and technology staff; and co-design meetings with UCSF, Chorus, and the FAB. Many ideas went through various iterations, with the FAB voice being taken into the highest consideration. FAB members provided different viewpoints and made decisions on design elements such as name and color scheme, as well as content elements such as specific questions and text language. Furthermore, having a technology partner with extensive experience in co-designing digital health technologies with diverse communities and ensuring equitable power sharing in the development of FostrSpace contributed a great deal to the success of the co-design space.

#### Ensure Mutual Benefit Among Co-Designers

The maintenance of reciprocal relationships during the co-design process is ideal, with all participants (academics, foster youth, clinicians, and developers) benefiting, whether through greater understanding of the self, community building, professional development, or furthering research. This relationship can allow for continued engagement in co-design and progression of the product, elucidation of honest and often sensitive or personal feedback, and novelty and creativity in the design process. Although FAB members held their own legitimacy as individuals with lived expertise in the foster care system, UCSF academics went through the process of creating staff positions for the FAB members, formalizing their roles and providing a professional title to add to their career trajectories and resume. FAB members also actively engaged in professional development opportunities such as presentations to community members, colleges, and donors to discuss the FostrSpace development. In hiring the FAB as staff, UCSF academics reaffirmed that FostrSpace would be a longer-term commitment and partnership. In turn, UCSF academics derived benefit from the stable consistency of FAB staff, who not only were committed to their work but also had the stability through financial support that allowed for continued engagement in the work. All co-designers were able to benefit by learning from one another (eg, UCSF academics and clinicians and FAB members learning about the vast capabilities of Chorus), building community, and having a safe space to share. All co-designers also benefited from building an emotional wellness app for foster youth—the FAB members by developing an app tailored to their needs and the needs of their fellow community members, UCSF academics by adding to the literature and development of foster youth interventions and services, and Chorus by partnering with another community to leverage participatory design practices.

### Limitations

The foster youth lived expertise is highly heterogeneous, and thus, there are limits to the generalizability found in the themes we derived. The cohort of co-design partners was diverse in ethnic origin, academic background, sexuality, and lived expertise; due to the small size of the FAB group (4 members), there are limitations to broader application as lived expertise in the foster care system can vary widely. Within the small group of FAB members, there was notable overlap in their lived expertise, especially due to the overrepresentation of students (all 4 FAB members were students in higher-education institutions, and 3/4, 75% were in the same college). The common profiles of tertiary education institutions and academic studies in the San Francisco Bay Area provide another limit to the generalizability of these findings to foster youth outside of the region. All FAB members had also been placed in kinship care, suggesting that barriers to mental health resources that are faced in other forms of foster care placement may not be adequately highlighted. Expanding the co-design process to include more diverse academic backgrounds (including youth who are not pursuing higher education), partners from regions beyond Northern California, and different forms of foster care placements appears to be a promising next step for improving intervention impacts and understanding how foster youth contribute to co-design processes.

### Conclusions

Lessons learned from this duoethnographic process can inform future researchers seeking to co-design digital health technologies with foster youth, as well as young people more broadly. The FAB’s determination of roles and level of participation underwent constant revision to accommodate and support FAB members as their lives developed over the course of the project. As suggested by Porche et al [5], by tailoring the nature and style of power sharing to the interests and thematic motivations of the co-design partners rather than just increasing the degree of power sharing and authority alone, a greater depth of engagement can be achieved. To this end, early and concurrent partnership with the intervention population to improve impact and ensure not only a satisfactory co-design process for the partners but also a tailored end product is essential [29]. Future research may consider the nuances of the themes derived from this duoethnography and devise methods to ensure that future co-design processes address those themes both through the actual co-design and in the developed intervention or app. Foster youth who have had extensive experiences with negative power dynamics in different spaces, for example, may benefit more from greater intentionality toward the cultivation of safety and empowerment in the co-design space than may be typically granted for other young people. Similarly, foster youth such as those who participated in this duoethnography process may have increased levels of negative experiences accessing resources and may be put off from the idea of trying to access future resources through a different platform; in this case, someone creating a resource bank may seek to provide more details on the resources being offered, tips and tricks for successful resource access, and potentially a difficulty rating or timescale indicating how much bandwidth a foster youth would have to commit in trying to access the resource.

Due to the diversity within the foster youth community, it may be difficult to develop a single plan that ensures adequate empowerment and recognition of each co-designer’s lived expertise in a co-design process, thereby reinforcing the need for active reflection throughout the co-design process. Continuous reflection co-design practices can be refined over time to meet the needs of a specific co-design team. The themes and subthemes gathered from the FostrSpace co-design team’s duoethnography may provide a helpful framework in starting to think about how to best serve foster youth throughout the co-design process. Co-design practices are vital for creating digital health technologies that are helpful, intuitive, and healing for their intended users. Co-design practices can draw out nuanced community knowledge and result in a thoughtful, vetted product. We call on researchers to continue the work of co-designing with young people and specifically foster youth, with a commitment to active and in-depth reflection throughout the co-design process.

## Abbreviations

FAB: FostrSpace Advisory Board
JJBH: Juvenile Justice Behavioral Health
TA: thematic analysis
UCSF: University of California, San Francisco
YPAR: youth participatory action research

## Data Availability

The datasets generated during and analyzed during this study is not publicly available due to ethical concerns about possible reidentification with this small study sample. Study metadata (eg, reflection questions and codebook) are available from the corresponding author on reasonable request.
